# First case report of bile leak from the duct of Luschka in a patient with mini-gastric bypass: The challenge of management

**DOI:** 10.1016/j.amsu.2018.09.018

**Published:** 2018-09-20

**Authors:** Houssam Khodor Abtar, Tarek Mostafa Mhana, Riad Zbibo, Mostapha Mneimneh, Antoine el Asmar

**Affiliations:** a Makassed General Hospital, Department of Surgery, Beirut, Lebanon; b Sahel General Hospital, Department of Radiology, Beirut, Lebanon

**Keywords:** Case report, Mini-gastric bypass, Duct of Luschka, Relaparoscopy

## Abstract

**Introduction:**

The incidence of Bile duct injury after laparoscopic cholecystectomy approaches 0.11%–1.4%. Ducts of Luschka are the second most common site of bile leaks. The rarity of these ducts with cases of anatomical alterations in the gastrointestinal tract such as mini-gastric bypass makes the management a challenging option.

**Presentation of case:**

Hereby we present a unique case of 28 year old female patient with mini-gastric bypass who had done uneventful cholecystectomy. Day 3 postoperatively patient complained of diffuse abdominal pain. Computed tomography showed free fluid in the abdomen. Liver enzymes were normal. Relaparoscopy showed leaking bile duct of Luschka, which was closed by surgical clips and drains left in the spaces. However bile leak continued for 4 weeks then stopped. Patient did well after all.

**Discussion:**

Endoscopic retrograde cholangiopancreatography with sphincterotomy played a crucial role for diagnosis and treatment of bile leaks with success rate near 94%. However no data were available using this method in a patient with Mini-gastric bypass procedure. Many authors have argued the role of relaparoscopy, but it is still an important way for adequate drainage and control of bile leakage. The only significant factor in determining clinical outcome in cases of non-surgical management is the type of bile duct injury.

**Conclusion:**

To the best of our knowledge, this is the first case report of bile leak from duct of Luschka after mini-gastric bypass treated successfully with relaparoscopy and drainage. Herein we will discuss all the available options of treatment and the challenge of it.

## Highlights

• The combination of Duct of Luschka in a patient with mini-gastric bypass is unique in the literature.
• The challenge of management especially endoscopic one puts a great pressure on both surgeons and gastroenterologists.
• NO single data available in the literature in doing ERCP by any method in a patient with mini-gastric bypass.

## 1 Introduction

This work has been reported in line with the SCARE criteria [1]. Ever since laparoscopic cholecystectomy became the gold standard for gallbladder removal, the experience in this procedure increased allowing surgeons to perform it in different contexts and pathologies [2]. However, a review of 1.6 million laparoscopic cholecystectomies showed an unchanged 0.3%–0.6% incidence of bile duct injury, with only 30% of injuries recognized at the time of operation [3]. Bile leak is a well-known and serious complication after cholecystectomy, being more common in its laparoscopic version. The cystic duct stump is by far the most common site of leak, followed by the ducts of Luschka, common bile duct, common hepatic duct and gallbladder bed. The most common presentation was bile in the surgical drain [4].

In 1863 Hubert Von Luschka described supravesicular ducts, branching form the right or common hepatic ducts, running in the submucosa of the posterior gallbladder wall with no communication with its lumen, not draining any liver parenchyma and ending blindly in their distal part [5]. According to the Strasberg classification, our patient was subject to type A injury where bile leak is due either to the cystic duct stump or Luschka duct, the latter in this case [6]. Mini-gastric bypass (MGB) was first reported by Rutledge in 1997. The result of the long intestinal loop, which is routinely bypassed at 200 cm (in both the alimentary and the biliopancreatic limbs) in association with restrictive component makes it safe and effective bariatric procedure with excess weight loss (EWL%) at 2 years ranges from 64.4% to 80% [7].

## 2 Presentation of case

**Fig. 1:**
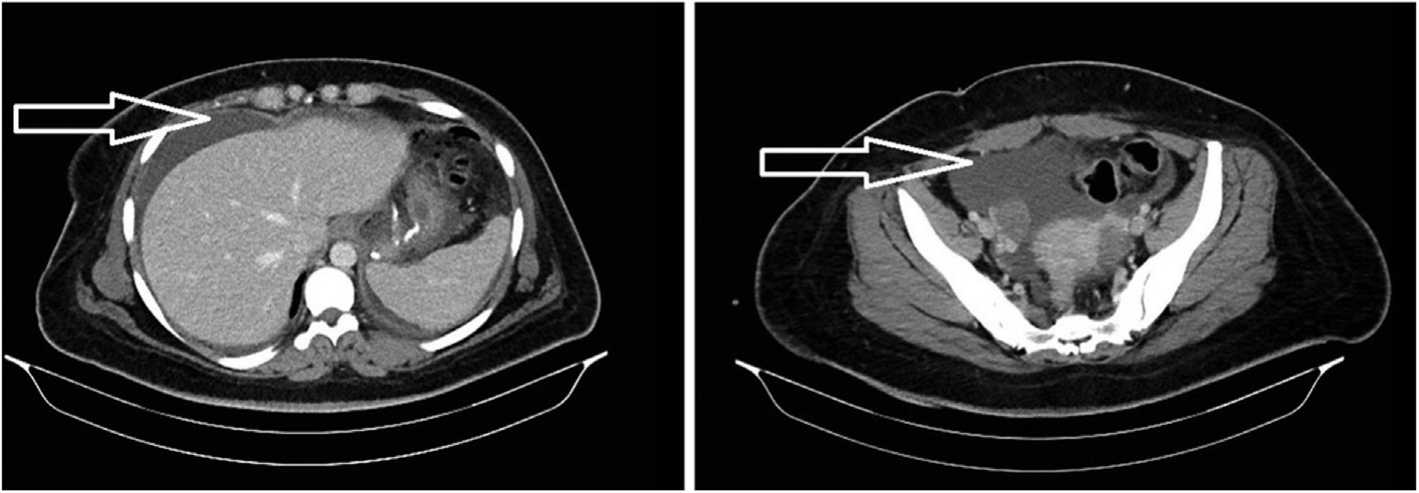
CT scan of abdomen showing fluid in the subhepatic and Douglas spaces (white arrow).

This is the case of a 28 year old female patient, nonsmoker, with BMI (body mass index) 37, and without any medical conditions or drug addiction, who underwent laparoscopic MGB procedure in our academic institution 6 months prior to presentation by an experienced bariatric and laparoscopic surgeon. Patient was re-admitted through emergency room with symptoms of biliary colic, ultrasound showed cholelithiasis without cholecystitis. She had uneventful laparoscopic cholecystectomy by the same surgeon. Three days later she presented with diffuse abdominal pain, nausea and vomiting, not associated with other gastrointestinal symptoms, nor with fever or jaundice. Laboratory work-up revealed a white blood cell count of 13000 with 84% neutrophils, positive C - reactive protein (CRP) value but no other abnormalities in the hepatic enzymes or bilirubin levels. An ultrasound of the abdomen was done showing fluid collection in the subhepatic space and in the Douglas pouch. Computed tomography (CT) scan confirmed the presence of fluid collection which was estimated to be around 1 L (Fig. 1), but no other abnormalities were detected. Reviewing the past surgical history of the patient, along with her presenting signs and symptoms, a decision for relaparoscopy was taken.

**Fig. 2:**
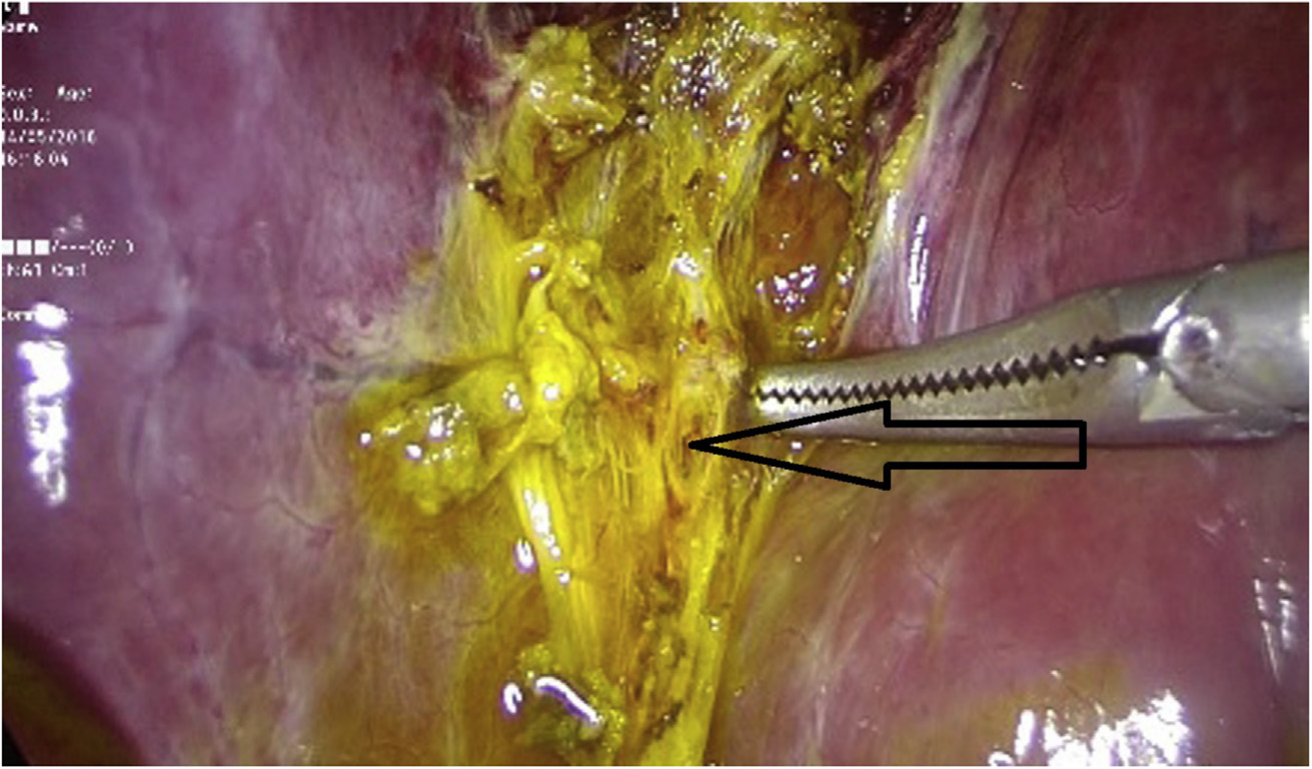
Bile leak from duct of Luschka in the liver bed (black arrow).

**Fig. 3:**
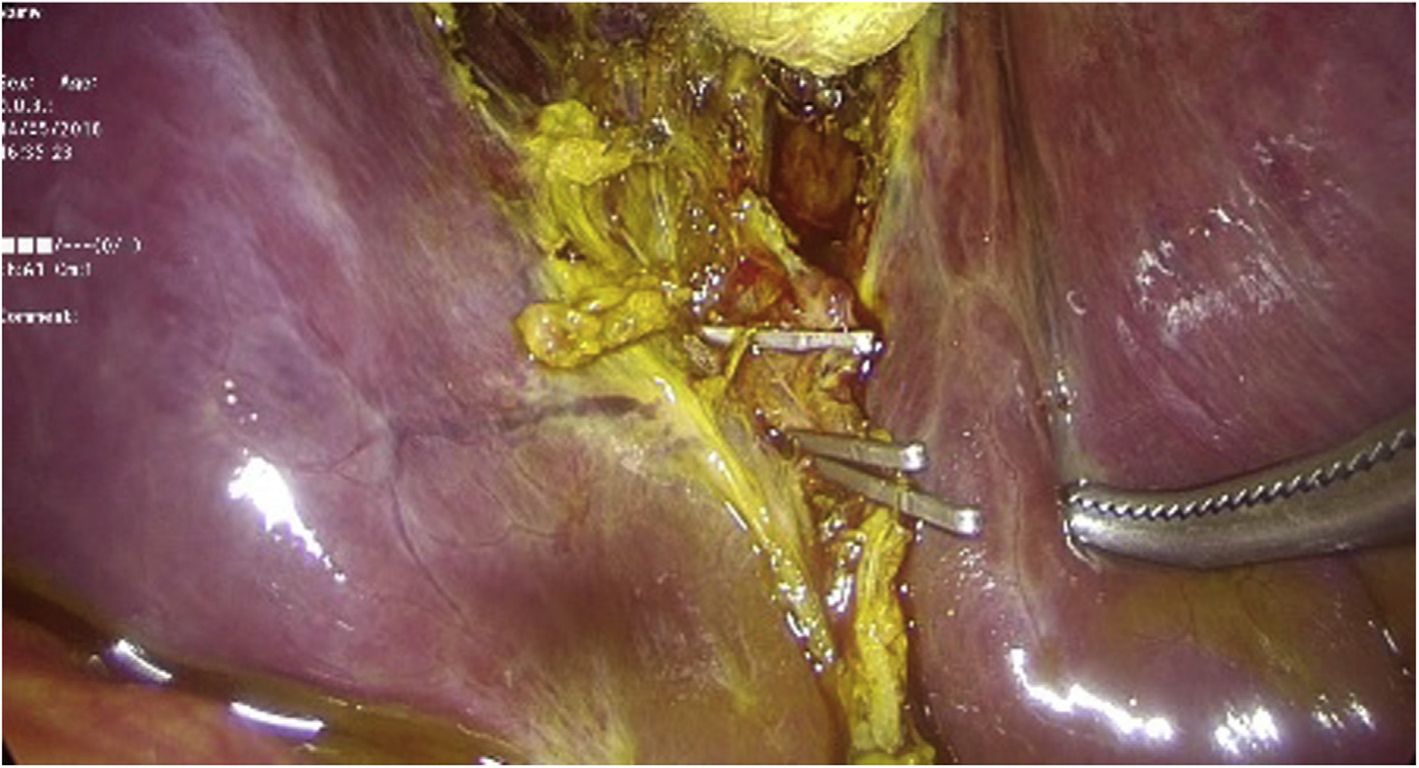
Intraoperative view of clips over the duct of Luschka.

Intraoperatively a large amount of bile was found in the abdomen, the gallbladder fossa was exposed and a leaking duct of Luschka was identified in the liver bed (Fig. 2), at the level of the triangle of “Calot” and around 3 cm away from the cystic duct. No other duct lumen was seen more distally. After exploration of all 4 quadrants making sure no other intra-abdominal pathologies were present, and removing all bile juice from the abdomen, the leaking Luschka duct was clipped (Fig. 3). Blake drains were inserted. Patient tolerated well the procedure and was transferred to the regular floor. The drain's output was serosities until the second day post operatively when it became bilious. The bile output was around 100 cc/day for 14 days, after which the quantity started to decrease and the color becoming clearer with time. The drain was kept and patient was taught how to empty it every day, until 4 weeks later, the drain's output was totally clear and near 5 cc/day. At that point the drains were removed. Follow-up visit 2 weeks later showed a healthy patient with no symptoms or sequelae.

## 3 Discussion

The prevalence of cholelithiasis, symptomatic or not, have been associated with all bariatric procedures and is maximal during the first 6 months where rapid weight loss occurs (more than 25% of original weight).The incidence of cholelithiasis in bariatric population has been estimated at about 13.6–47.9%. However the reported incidence of symptomatic cholelithiasis has been estimated to be from 2.9 to 14.7%. Not much data is available for MGB but Mishra et al. reported incidence of 12.7% after MGB [8].

The fact that laparoscopic cholecystectomy was a minimally invasive surgical technique compared to its open counterpart, the need for a minimally invasive technique to treat its complication was crucial. With the advances in endoscopic procedures, skills and expertise of gastroenterologists, endoscopic retrograde cholangiopancreatography (ERCP) revealed itself as an efficient and safe modality for diagnosis and treatment of bile leak after laparoscopic cholecystectomy [4]. Sphincterotomy allows lowering of the transpapillary pressure gradient through ampullary orifice, and stenting, if feasible, allows bridging of the leak site thus healing of the injured part. Success rate was near 94% in these interventions [9]. Further studies handled the potential of percutaneous drainage only. They successfully managed to treat bile leak in 14 out of 17 patients with drainage only. They concluded that such a method can be safely implemented in cases where no jaundice is present, and in those where bile output decreases after catheterization [10].

All conducted studies regarding ERCP after bariatric surgery were based on Roux-en-Y gastric bypass (RYGB) cases [11]. First, balloon-assisted enteroscopy or double-balloon enteroscopy, was effective in treating bile leak from Luschka's duct in a patient with RYGB [12]. Schreiner et al. reported a significantly higher rate of therapeutic success in patients having a total limb length less than 150 cm compared with those with a total limb length of 150 cm or longer (88% vs. 25%, respectively). The estimated success rate of ERCPs was approximately 60% in most of the literature [13]. However no data were available for MGB procedures, where such a technique would be facing the challenge of a 200 cm limb. Second, Laparoscopy-assisted transgastric ERCP (LA-ERCP) revealed effective, safe and reliable method of management and can be performed by a minimally invasive approach with technical success approaching (100%) [14]. LA-ERCP was found superior than balloon enteroscopy ERCP especially in patients with length of Roux limb plus biliopancreatic limb more than 150 cm but no single case was ever performed in the literature. Choi et al. showed that ERCP via gastrostomy is hindered by the gastrostomy maturation delay and a higher morbidity [15]. Third, the latest approach consisted of endoscopic ultrasound-directed transgastric ERCP, where access to the excluded stomach is done through the creation of a gastro-gastric fistula tract, covered with a metallic stent that allowed the antegrade passage of a duodenoscope [16]. However, no cases after MGB procedure were reported.

Owing to our patient's clinical status, our institutions facilities and our technical context, we decided to undergo relaparoscopy for the patient. Relaparoscopy remains one of the options in case bile leak is suspected. Many authors have argued the importance of relaparoscopy with bile leakage control reaching up to 90% [17]. In the context of bile leak, the type of bile duct injury according to the Strasberg classification, was the only statistical significant factor in determining clinical outcome in cases of non-surgical management [18]. Time-to-diagnosis interval is a very important factor as well. Many authors have argued the fact that delayed detection of bile duct injury may lead to increased morbidity, increased severity of the injury, treatment failure and even death [19]. Injuries recognized intraoperatively require immediate primary repair with a clip or ligature and drainage for type A and D lesions. Common bile duct (CBD) transection or aberrant duct (B, C and E types) requires hepatico-jejunostomy as a best option. In case of postoperative diagnosis, (<6 weeks, time to form stenosis), all types lesions are treated with percutaneous drainage of abscess or biloma followed by ERCP with stent application. However complex lesion (B, C or E) should be treated by biliodigestive repair 6–8 weeks after drainage and ERCP so that the inflammation process subsided. Because injuries of late postoperative diagnosis (≥6 weeks) are mainly ischemic stenosis, treatment is controversial. ERCP with dilatation and stenting should be tried, however biliodigestive anastomosis with liver resection or transplantation could be necessary [20].

## 4 Conclusion

It is interesting to consider one of the abovementioned approaches in our case. But the question raised would be, what is the degree of success in MGB in a time all these procedures were done in Roux-en-Y gastric bypasses? What are the determining factors to undergo a conservative, endoscopic, method versus the more aggressive option, relaparoscopy? Many questions are still pending regarding the safety and effectiveness of endoscopic procedures in MGB context with bile leak or choledocholithiasis. Relaparoscopy, drainage and conservative management afterwards were successful as shown by our case. Will the leap towards endoscopic-based treatment happening anytime soon. Hereby we present the first reported case in the literature and there is no doubt that this case will add an informative value to the surgical literature in terms of diagnosis and management this pathology.

## Ethical approval

None.

## Sources of funding

None.

## Author contribution

Writing of the manuscript, study design and interpretation of the data: Houssam Khodor Abtar; Interpretation of radiological results and article review: Tarek Mostafa Mhana; Article review and interpretation of the data: Riad Zbibo; Study design and article review: Mostapha Mneimneh; Writing of the manuscript, study design and interpretation of the data: Antoine el Asmar.

## Conflicts of interest

The author declares no conflict of interest.

## Research registration number

researchregistry2218.

## Guarantor

Houssam Khodor Abtar.
